# Psilocybin in the treatment of obsessive-compulsive disorder: What do we know so far?

**DOI:** 10.1192/j.eurpsy.2021.1114

**Published:** 2021-08-13

**Authors:** N. Descalço, A.B. Medeiros, C. Fernandes Santos, G. Borges

**Affiliations:** Psychiatry And Mental Health Department, Hospital Garcia de Orta, E.P.E., Almada, Portugal, Portugal

**Keywords:** Obsessive-Compulsive disorder, Psilocybin, Hallucinogens

## Abstract

**Introduction:**

Psilocybin is a naturally occurring plant alkaloid in mushrooms and a prodrug of psilocin. It is a serotonin receptor (5-HT2A) agonist and known psychedelic, with similar hallucinatory properties to lysergic acid diethylamide (LSD). It has been identified as a safe and effective option in treatment-resistant depression. Literature focus mainly on its use on depressive but its interest in other psychiatric disorders such as obsessive-compulsive disorder (OCD) has grown.

**Objectives:**

To review the clinical evidence for the use of hallucinogens such as psilocybin in OCD.

**Methods:**

Non-systematic review of literature found on PubMed/MEDLINE, Web of Science and Google Scholar, using the keywords “obsessive-compulsive disorder”, “psilocybin” and “hallucinogens”. Articles may include clinical trials, case report or case series. Articles found were admitted according to their relevance for the topic in review; only articles in English were included. Ongoing research trials on this topic were checked on ClinicalTrials.gov.

**Results:**

So far, only one open-label non-randomized study directly assessed the effects of psilocybin on OCD patients that found acute reductions of obsessive-compulsive symptoms. Case reports of patients improving with off-label use of psilocybin are reported. There are two ongoing phase I research trials, aiming to explore the effect of the substance on symptomatology, hypothesizing that psilocybin will normalize cerebral connectivity and thus correlate with clinical improvement.

**Conclusions:**

More research to establish the usefulness of psilocybin in OCD patients is needed; the collected data is encouraging are there may be a role for its use on this disorder.

